# Leveraging Disruptions to Create an Equitable, Age-Friendly, Learning Health System

**DOI:** 10.1093/geroni/igab046.1124

**Published:** 2021-12-17

**Authors:** Eric Lenze, Brian Carpenter, Nancy Morrow-Howell, Beth Prusaczyk

**Affiliations:** 1 Washington University School of Medicine in St. Louis, Saint Louis, Missouri, United States; 2 Washington University in St. Louis, Saint Louis, Missouri, United States

## Abstract

In a learning health system, the system’s own data and the experiences of its workforce are integrated with external evidence to provide better care. In an age-friendly health system, core principles of age-friendly care are integrated into every point in the system. Disruptions caused by the COVID-19 pandemic, and the innovations that addressed them, present an opportunity to discuss how these two frameworks may be combined and leveraged to transform care for older adults. We will present examples of pandemic-related disruptions, including rapid changes in how patients and providers move within and between facilities and the significant toll on healthcare workers’ mental health. We will also highlight innovative solutions to these disruptions that could transform healthcare systems. Critical to these points is a discussion of how these disruptions have disproportionately impacted healthcare workers and patients of color and how the innovations must be implemented using an anti-racist, health equity lens.

